# A Single Nucleotide Polymorphism in the *Stromal Cell-Derived Factor 1* Gene Is Associated with Coronary Heart Disease in Chinese Patients

**DOI:** 10.3390/ijms150611054

**Published:** 2014-06-19

**Authors:** Lei Feng, Shi-Yan Nian, Ying-Lu Hao, Wen-Bo Xu, Dan Ye, Xing-Feng Zhang, Dan Li, Lei Zheng

**Affiliations:** 1 Department of Laboratory Medicine, Nanfang Hospital, Southern Medical University, Guangzhou 510515, China; E-Mail: fengleii@kmmu.edu.cn; 2 Department of Laboratory, People’s Hospital of Yuxi City, Yuxi 653100, China; E-Mails: xwb103196@sina.com (W.-B.X.); yedan16@163.com (D.Y.); zxf1027zxf@163.com (X.-F.Z.); lidan1224@163.com (D.L.); 3 Intensive Care Unit, People’s Hospital of Yuxi City, Yuxi 653100, China; E-Mail: lili580923@kmmu.edu.cn; 4 Department of Cardiology, People’s Hospital of Yuxi City, Yuxi 653100, China; E-Mail: haoyinglu@kmmu.edu.cn

**Keywords:** single nucleotide polymorphism (SNP), *stromal cell-derived factor 1* (*SDF-1*) gene, coronary heart disease (CHD), Chinese patients

## Abstract

Coronary heart disease (CHD) is highly prevalent globally and a major cause of mortality. Genetic predisposition is a non-modifiable risk factor associated with CHD. Eighty-four Chinese patients with CHD and 253 healthy Chinese controls without CHD were recruited. Major clinical data were collected, and a single nucleotide polymorphism (SNP) in the *stromal cell-derived factor 1* (*SDF-1*) gene at position 801 (G to A, rs1801157) in the 3'-untranslated region was identified. The correlation between rs1801157 genotypes and CHD was evaluated by a multivariate logistic regression analysis. The allele frequency in the CHD and control groups was in Hardy-Weinberg equilibrium (HWE) (*p* > 0.05). The frequency of the GG genotype in the CHD group (59.5%) was significantly higher than that in the control group (49.8%) (*p* = 0.036). A number of variables, including male sex, age, presence of hypertension, and the levels of low-density lipoprotein cholesterol (LDL-C), high-density lipoprotein cholesterol (HDL-C), triglycerides (TG), uric acid, and total bilirubin, were associated with CHD in a primary univariate analysis. In a multivariable logistic regression analysis, the GG genotype (GG:AA, odds ratio (OR) = 2.31, 95% confidence interval (CI) = 1.21–5.23), male sex, advanced age (≥60 years), presence of hypertension, LDL-C level ≥ 3.33 mg/dL, HDL-C level < 1.03 mg/dL, and TG level ≥ 1.7 mg/dL were independent risk factors for CHD.

## 1. Introduction

Coronary heart disease (CHD) is caused by obstruction of epicardial coronary artery that supplies blood and oxygen to the heart [1]. The narrowing of these small arteries originates from plaque buildup in the coronary arteries—a condition called atherosclerosis [1]. Intravascular cholesterol-laden plaque deposits in blood vessel walls cause unique local hydrodynamic characteristics, and as individuals age the plaque burden increases, inflaming the vessel walls and raising the risk of blood clot formation and heart attack [2]. Plaques release chemicals that make the inner walls of blood vessels sticky [3]. This can aggravate atherosclerosis by causing various substances, including inflammatory cells, lipoproteins, and calcium, in the bloodstream to stick to the inside of the vessel wall [1,2,3]. In some cases, a blood clot may completely cut off the blood supply to the heart muscle, causing a heart attack. Thus, CHD is a major health threat to people around the world [1,2,3].

Over the last few decades, our understanding of the pathophysiology of CHD has evolved remarkably [4]. Atherosclerosis is now recognized as an inflammatory disorder with a complex set of interacting risk factors, including cells of the artery wall and the blood and molecular messages that they exchange [1,2,3,4]. Modifiable risk factors associated with CHD include smoking, high blood pressure, high blood cholesterol or dyslipidemia, high blood sugar, coagulation factor VII, lack of exercise, stress, obesity, and a diet rich in saturated fats and low in antioxidants [5,6]. Non-modifiable risk factors associated with CHD include advanced age, male sex, and genetic predisposition [5,6,7,8]. Single nucleotide polymorphisms (SNPs) are widely studied and important genetic factors that can affect an individual’s genetic predisposition for various diseases [9]. Genome-wide association studies (GWAS) have identified more than 100 common gene variants that are associated with the risk of CHD as reported in the National Human Genome Research Institute catalog [10].

Stromal cell-derived factor 1 (SDF-1), also known as C–X–C motif chemokine 12 (CXCL12), is a chemokine that in humans is encoded by the *CXCL12* gene located at q11.21 on chromosome 10 [11,12]. SDF-1 may activate T-lymphocytes and is induced by proinflammatory stimuli such as lipopolysaccharide, tumour necrosis factor (TNF), or interleukin-1 (IL-1). SDF-1 activates C–X–C chemokine receptor type 4 (CXCR4) to induce a rapid and transient rise in the level of intracellular calcium ions and chemotaxis [11,12,13,14]. SDF-1 may also bind to atypical chemokine receptor 3, which activates the β-arrestin pathway and acts as a scavenger receptor for SDF-1 [11,12,13,14]. SDF-1-β and -α show reduced chemotactic activity. Thus, SDF-1 plays an important role in host inflammatory responses [11,12,13,14]. An SNP at position 801 (G to A, rs1801157) in the 3'-untranslated region (3'-UTR), whose an allele is regarded as a target of *cis*-acting factors, has been shown to up-regulate the expression of CXCL12 [15]. Studies have shown that rs1801157 is associated with susceptibility to blast invasion in acute myelogenous leukemia [16], sporadic prostate cancer [15], and breast cancer [17,18]. In summary, SDF-1 possesses diverse physiological and biochemical functions *in vivo* and SNPs in the SDF-1 gene play various roles in many pathophysiologic processes. Since inflammation is believed to participate in the local, myocardial, and systemic complications of atherosclerosis, it is necessary to explore whether there is a correlation between SDF-1 and CHD. In this study, we focused on the SNP rs1801157 in the 3'-UTR of the SDF-1 gene. Specifically, the prevalence of the rs1801157 genotype in Chinese CHD patients and healthy (non-CHD) Chinese controls was evaluated.

## 2. Results

### 2.1. Description of the Study Population

We recruited 84 patients with CHD and 253 healthy controls without CHD in this study. The proportion of males and presence of hypertension were significantly greater in the CHD group than in the control group. The age and serum levels of LDL-C, uric acid, and total bilirubin in the CHD group were significantly higher than those in the control group. The serum level of HDL-C in the CHD group was significantly lower than that in the control group (Table 1).

**Table 1:** Demographics of the study population.

Variables	Without CHD (*N* = 253)	With CHD (*N* = 84)	*p*
Sex: male (%)	152 (60.1)	66 (78.6)	0.0023
Age (years)	45 (26–60.3)	55 (45.8–71)	<0.001
Presence of hypertension	26 (10.3)	52 (61.9)	<0.001
LDL-C (mg/dL)	2.28 (1.81–2.62)	2.60 (1.90–3.34)	0.026
HDL-C (mg/dL)	1.16 (0.86–1.35)	1.02 (0.99–1.34)	0.003
TG (mg/dL)	1.80 (1.05–2.88)	1.61 (1.19–2.01)	0.102
Uric acid (mg/dL)	253 (311–382)	310 (357–420)	<0.001
Total bilirubin (mg/dL)	5.9 (7.80–10.30)	7.23 (9.90–11.80)	0.033

Skewed data are presented as the median (interquartile range) and categorical data are presented as the number (%). Abbreviations: HDL-C, high-density lipoprotein cholesterol; LDL-C, low-density lipoprotein cholesterol; TG, triglycerides. Differences in the baseline characteristics of the four groups were examined using the Kruskal-Wallis H test, one-way ANOVA, Fisher’s exact test, or χ^2^ tests according to the distribution of the data.

### 2.2. Genotype and Allele Distributions in the Case and Control Populations

The genotype distribution of SNP rs1801157 in the control group was as follows: 120 (49.8%), 111 (43.9%), and 22 (6.3%) individuals possessed the GG, GA, and AA genotypes, respectively (Table 2). The genotype distribution of SNP rs1801157 in the CHD group was as follows: 50 (59.5%), 30 (35.7%), and 4 (4.8%) individuals possessed the GG, GA, and AA genotypes, respectively (Table 2). The AA genotype was rare in both the CHD and control groups, while the frequency of the GG genotype in the CHD group was significantly higher than that in the control group (*p* = 0.036) (Table 2). The Hardy-Weinberg equilibria of the genotypes were evaluated using dedicated software [19]. The allele frequency in the CHD and control groups was in Hardy-Weinberg equilibrium (HWE) (*p* > 0.05), suggesting that the genotype frequencies in the case and control populations remained constant in terms of their genetic background. Regarding allele distribution, the frequencies of G and A were 69.4% and 30.6%, respectively, in the control group; while the frequencies of G and A were 77.4% and 22.6%, respectively, in the CHD group; overall, the frequency of the G allele was significantly higher in the CHD group than in the control group (Table 2). Although the frequency of the A allele was significantly lower in the CHD group than in the control group, its homozygote (AA) or heterozygote (AG) displayed no significant difference in distribution (Table 2).

**Table 2:** Distributions of genotypes and alleles in the case and control populations.

Genotypes	Without CHD (*N* = 253)	With CHD (*N* = 84)	*p*-Value for Distribution
G/G	120 (49.8)	50 (59.5)	0.036
G/A	111 (43.9)	30 (35.7)	0.118
A/A	22 (6.3)	4 (4.8)	0.176
*p* value for HWE	>0.05	>0.05	
**Alleles**	**Without CHD (*N* = 253)**	**With CHD (*N* = 84)**	***p*-Value for Distribution**
G	351 (69.4)	130 (77.4)	0.027
A	155 (30.6)	38 (22.6)	

Genotypes or alleles are presented as the frequency (%). Abbreviations: CHD, coronary heart disease; HWE, Hardy-Weinberg equilibrium.

### 2.3. Associations of rs1801157 Genotype with Coronary Heart Disease (CHD)

Our primary statistical analysis showed that male sex, age, presence of hypertension, and levels of low-density lipoprotein cholesterol (LDL-C), high-density lipoprotein cholesterol (HDL-C), triglycerides (TG), uric acid, and total bilirubin were associated with CHD (Table 1). Genotype and allele distribution analyses showed that genotype GG of rs1801157 was also associated with CHD (Table 2). To evaluate whether these factors were independently associated with CHD, a multivariable logistic regression analysis was conducted. Sex, age, presence of hypertension, and the levels of LDL-C, HDL-C, TG, uric acid, and total bilirubin were all converted into binary variables and added to multivariable logistic regression models together with the various rs1801157 genotypes. As shown in Table 3, seven factors remained as independent risk factors for CHD: the GG compared with the AA genotype, male sex, age ≥ 60 years, presence of hypertension, LDL-C level ≥ 3.33 mg/dL, HDL-C level < 1.03 mg/dL, and TG level ≥ 1.7 mg/dL (to see all of the ORs and 95% CIs, see Table 3).

## 3. Discussion

Among the genetic factors associated with disease, SNPs have a major influence on individual susceptibility to various diseases [20]. As of 6 May 2014, 1912 publications covering 13,270 SNPs found to be correlated with CHD in GWAS were cataloged in the National Human Genome Research Institute database [21]. The genes described in these studies were distributed throughout chromosomes 1 to 22. Efforts at collectivization such as this will contribute to the production of a comprehensive list of SNPs associated with CHD and will promote the development of personalized medicine [22,23,24,25]. It is worth noting that although GWAS offer a tremendous amount of information on SNPs, they cannot identify all of the SNPs involved in a single action. Indeed, rs1801157 was not included in any of the above reports.

**Table 3:** Factors associated with the presence of coronary heart disease (CHD) in the multivariate analysis.

Factor	Category	OR	95% CI
rs1801157 genotypes	G/G	2.31	1.21–5.23
	G/A	0.59	0.21–0.56
	A/A	1.00	
Hypertension	Presence	3.12	1.78–5.13
HDL-C	<1.03 mg/dL	0.43	0.21–1.32
LDL-C	≥3.33 mg/dL	1.33	1.01–2.98
TG	≥1.7 mg/dL	1.75	1.24–5.13
Sex	Male	3.12	1.54–4.32
Age	≥60 years	2.11	1.09–3.43

Abbreviations: HDL-C, high-density lipoprotein cholesterol; LDL-C, low-density lipoprotein cholesterol; TG, triglycerides; OR, odds ratio; CI, confidence interval.

SDF-1 possesses diverse physiological and biochemical functions *in vivo*, and SNPs in the SDF-1 gene play various roles in many pathophysiologic processes [13,14,15,16,17,18]. Among the SNPs in the SDF-1 gene, an SNP at position 801 (G to A, rs1801157) in the 3'-UTR is the most studied. As of 6 May 2014, there were 34 reports on rs1801157 in the SNP database of the National Center for Biotechnology Information [26]. The *CXCL12-3'A* variant has been demonstrated to be associated with multiple myeloma, acute lymphoblastic leukemia, various tumors, and the outcome of human immunodeficiency virus infection [13,14,15,16,17,18,26]. A meta-analysis of 17 studies that included 3048 cancer patients and 4522 controls showed that variant genotypes were associated with a significantly increased risk of all cancer types (OR = 1.38, 95% CI = 1.18–1.61 for GA *versus* GG, and OR = 1.36, 95% CI = 1.17–1.59 for GA/AA *versus* GG) [27]. In our study, the AA genotype of rs1801157 was shown to be more rare than in most previous studies. This might be explained by racial differences between the subjects. Furthermore, in this study, the GG genotype was associated with the presence of CHD. This finding may provide a new molecular genetic mechanism for rs1801157 in human disease. We performed an association analysis using a single allele and found that the A allele was protective for our population; however, we did not include this finding in our current results because it might be due to the significant contribution of the G allele to CHD morbidity.

In processing the primary data, TC, LDL-C and apolipoprotein B (apoB), and HDL-C and apolipoprotein A-I (apoA-I) displayed collinearity, together with serum lipid metabolism and the preliminary diagnostics. In our subsequent statistical analysis, we adopted LDL-C (representing TC, LDL-C, and apoB) and HDL-C (representing HDL-C and apoA-I) as variables to represent serum lipids. Other serum biochemical elements, including homocysteine, were excluded because no impact on the correlation between the SNPs of rs1801157 and CHD was found during processing of the primary data. Our multivariable logistic regression analysis demonstrated that male sex, advanced age (≥60 years), presence of hypertension, LDL-C level ≥ 3.33 mg/dL, HDL-C < 1.03 mg/dL, and TG ≥ 1.7 mg/dL were independent risk factors for CHD, while, although uric acid and total bilirubin were also associated with CHD in our primary single variable statistical analysis, these two factors were removed from the multivariable logistic regression analysis, likely due to the interaction between these and other factors in the pathophysiology of CHD.

The CXCL12–CXCR4 axis plays multiple roles in peripheral and central organs. During embryogenesis, it directs the migration of hematopoietic cells from the fetal liver to bone marrow and the formation of large blood vessels [28]. In adulthood, CXCL12 plays an important role in angiogenesis by recruiting endothelial progenitor cells from the bone marrow through a CXCR4-dependent mechanism [28]. Thus, although we did not perform a functional assessment of rs1801157 genotypes, it is possible that an SNP in the *SDF-1* gene generates a particular phenotype of CXCL12 and contributes to the pathophysiologic process of CHD.

This study represents a cross-sectional SNP analysis since CHD is more frequent in elderly populations, making it difficult to construct an age-matched control group, while our multivariate model included most of the previously reported CHD risk factors plus rs1801157 polymorphisms, and the GG genotype was demonstrated to be an independent risk factor for CHD. The uncertainty of the ORs arising from the study design might be resolved in a large-scale population-based study.

The limitations of this study include: First, the lack of any functional assessment of rs1801157 genotypes; second, comparing to genome wide association study, our research only focused on one SNP site and performed on small population; third, since we have not replicated this study in a population with different ethnicity, we are not sure if the association between rs1801157 and CHD is Chinese specific.

## 4. Methods

### 4.1. Patients

The Review Board of People’s Hospital of Yuxi City (Yuxi, China) approved this study (approval number: YNYXH2010-0012; 1 May 2010). Written informed consent was obtained according to the guidance of the Chinese National Ethics Regulation Committee. Participants were simultaneously informed of their right to repeal consent by themselves or their kin, caretakers, or guardians.

To assess genetic polymorphisms related to CHD, we recruited 84 ethnic Han Chinese patients (66 males and 18 females; age range, 45.8–71 years; median age, 55 years) with CHD who were unrelated consecutive inpatients at People’s Hospital of Yuxi City between September 2010 and December 2012. To obtain an estimate of the genetic distribution of the reference allele in the general population, we also randomly obtained DNA samples from 253 healthy individuals with no history of CHD who visited the People’s Hospital of Yuxi City (152 males and 101 females; age range, 26–60.3 years; median age, 45 years). The 253 healthy controls did not have a history of chronic disease, autoimmune disease, or cardiovascular disease.

Patients were diagnosed with CHD according to American Heart Association guidelines [29]. All patients were confirmed by the obstruction of at least 1 large epicardial coronary artery by atheromatous plaque using coronary angiography. Patients who met the exclusion criteria will be exclude from this study: alcohol abuse, diabetes, a history of smoking, chronic lung disease, xanthelasma, and evidence of noncoronary atherosclerotic disease. Hypertension was defined as a systolic pressure >140 mmHg or a diastolic pressure >90 mmHg. The following clinical parameters were obtained for each subject at the time of whole blood collection: age, sex, and the levels of TG, HDL-C, LDL-C, uric acid, and total bilirubin.

### 4.2. Polymorphism Genotyping

Genomic DNA was extracted from 100 µL of whole blood using a QIAamp DNA Blood Mini Kit (QIAGEN, Gaithersburg, MD, USA). The extracted DNA was dissolved in 20 µL of 10 mM Tris–HCl buffer (pH 8.0) containing 1 mM EDTA. All DNA samples were stored at −30 °C until use.

**Figure 1:**
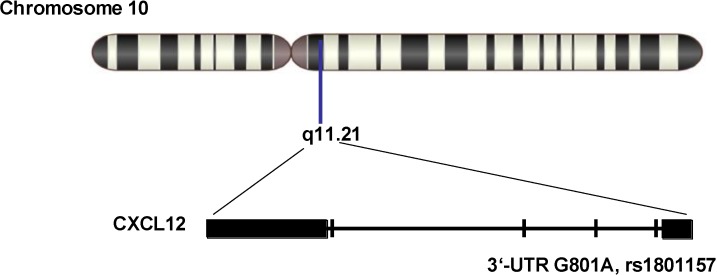
Diagram of rs1801157. *SDF-1*, also known as *CXCL12*, is encoded by the *CXCL12* gene located at q11.21 on chromosome 10. rs1801157 is located at position 801 in the 3'-UTR.

Genetic polymorphisms in *SDF-1* were identified using the MassARRAY^®^ system (Sequenom, San Diego, CA, USA) according to the manufacturer’s user guide. We used the primers F (5'-CAGTCAACCTGGGCAAAGCC-3') and R (5'-CCTGAGAGTCCTTTTGCGGG-3') (GenBank accession number: L36033) to amplify the specific fragment that covers rs1801157 (Figure 1). A 293-bp *SDF-1* fragment was PCR-amplified using the extracted genomic DNA as template. PCR was performed using HiFiFast DNA polymerase (Biovisualab Inc., Shanghai, China). The thermocycling conditions were as follows: 94 °C for 5 min, followed by 35 cycles of 94 °C for 10 s, 55 °C for 30 s, and 72 °C for 30 s. To verify the size of the PCR product, amplicons were visualized on 12.5% polyacrylamide gels with appropriate size markers.

The MassARRAY^®^ system is based on single base primer extension technology. MassARRAY^®^ technology uses matrix-assisted laser desorption ionization time-of-flight mass spectrometry to measure the mass of the extension product(s) directly and to correlate the detected mass with a specific genotype. The extension primer sequence was 5'-GCCCTCCCAGAAGAGGCAGACC-3'. For details on the protocol, please refer to “SNP Genotyping Using the Sequenom MassARRAY^®^ iPLEX Platform (http://www.sequenom.com/)”.

### 4.3. Statistical Analyses

Associations between the clinical parameters (age, sex, TG, HDL-C, LDL-C, uric acid, total bilirubin, and rs1801157 genotype) and the presence of CHD were evaluated using Student’s *t*-tests, Mann-Whitney U tests, and χ^2^ tests. Associations between the genotype at each locus and the presence of CHD were evaluated using χ^2^ tests. The Cochran-Armitage test was used to test for trends. Possible confounding effects among the variables were adjusted using a multivariate logistic regression model, and odds ratios (ORs) and 95% confidence intervals (CIs) were calculated. *p* < 0.05 was considered significant in the two-tailed tests. Hardy-Weinberg equilibria of the alleles at each individual locus were evaluated using software designed to detect Hardy-Weinberg equilibrium (HWE) [30].

A multivariate logistic regression model was used to calculate the statistical power required to detect the contribution of an SNP to the risk of CHD while including other known risk factors for CHD. SNP status was defined as *X* =0, 1, or 2, corresponding to homozygous for an allele, heterozygous, or homozygous for a different allele, respectively [31]. The required sample size (*N*) for the multivariate logistic regression analysis was calculated as reported previously. When the effect size of an SNP was assumed to be 0.69, which corresponded to an OR of 2, the required sample size was calculated to be 75% or 100% for a statistical power of 80% or 90%, respectively. Based on these calculations, our sample size was sufficient for conditions in which the OR of an SNP exceeded 2.

## 5. Conclusions

The GG genotype of rs1801157 in *SDF-1* is an independent risk factor for CHD in Chinese populations.
